# Apatinib Treatment in Metastatic Gastrointestinal Stromal Tumor

**DOI:** 10.3389/fonc.2019.00470

**Published:** 2019-06-11

**Authors:** Zhaolun Cai, Xin Chen, Bo Zhang, Dan Cao

**Affiliations:** ^1^Department of Gastrointestinal Surgery, West China Hospital, Sichuan University, Chengdu, China; ^2^Department of Abdominal Oncology, Cancer Center of West China Hospital, Sichuan University, Chengdu, China

**Keywords:** apatinib, gastrointestinal stromal tumors, GISTs, metastatic, vascular endothelial growth factor receptor

## Abstract

**Background:** Gastrointestinal stromal tumors (GISTs) are the most common mesenchymal tumors of the gastrointestinal tract. The clinical management of patients with metastatic GISTs is exceptionally challenging due to their poor prognosis. Apatinib is a multiple tyrosine kinase inhibitor. Here, we present the unique case with metastatic GISTs who derived clinical benefit from apatinib following the failure of imatinib and sunitinib.

**Case presentation:** A 57-year-old man was admitted to our hospital diagnosed with metastatic and recurrent GISTs following surgical resection. Fifty-four months after the first-line imatinib treatment, he developed progressive disease and then was treated with cytoreductive surgery combined with imatinib. Disease progression occurred after 7 months. He then received second-line sunitinib and achieved a progression-free survival of 11 months. Apatinib mesylate was then administered. Follow-up imaging revealed a stable disease. Progression-free survival following apatinib therapy was at least 8 months. The only toxicities were hypertension and proteinuria, which were both controllable and well-tolerated.

**Conclusions:** Treatment with apatinib provides an additional option for the treatment of patients with GISTs refractory to imatinib and sunitinib.

## Introduction

Gastrointestinal stromal tumors (GISTs) are the most common mesenchymal neoplasms of the gastrointestinal tract (1). Most GISTs harbor activating mutations in either gene encoding KIT or platelet-derived growth factor receptor-α (PDGFRA) (2, 3), which are type III receptor tyrosine kinases (4). After the identification of activating mutations, tyrosine kinase inhibitors (TKIs) are used for GISTs (5), which also significantly improved prognosis of patients with metastatic or recurrent disease (6). Imatinib mesylate is recommended as initial therapy based on drug activity and drug tolerability according to the National Comprehensive Cancer Network (NCCN) guideline for advanced GISTs (7). For patients with metastatic or unresectable GISTs after the failure of imatinib, sunitinib is considered (7). However, resistance to sunitinib eventually develops in most patients, after a median of 6–9 months (8). Although regorafenib can significantly improve survival as the third-line treatment, median progression-free survival (PFS) was only 4.8 months. Therefore, the development of efficacious and safe therapies is required for the treatment of metastatic or unresectable GISTs after the failure of imatinib and sunitinib.

Apatinib (Hengrui Pharmaceutical Co., Ltd., Shanghai, China) is a multiple TKI and targets vascular endothelial growth factor receptor 2 (VEGFR2), PDGFRβ, c-Kit, and c-src (9–11). Apatinib has been proved to be effective and safe in several solid tumors. However, there is no report for apatinib in treating GISTs today. Here, we present a case with metastatic GISTs that was effectively treated by apatinib following the failure of imatinib and sunitinib, demonstrating the potential efficacy of apatinib in the treatment of metastatic or unresectable GISTs. To the best of our knowledge, this is the first case of metastatic GISTs that responds to apatinib.

## Case Presentation

A 57-year-old asymptomatic man was found to have multiple intestinal masses by computed tomography (CT) done as part of his routine medical examination in December 2011. Surgical resection (R0) was performed in December 2011. The resected specimen consisted of a mass measuring 10.0 ×10.0 cm in maximal diameter. Final pathologic diagnosis revealed a high-risk GIST according to the Armed Forces Institute of Pathology (AFIP) criteria (12). The patient was not treated with adjuvant treatment after surgery in the local hospital because he had difficulty paying for adjuvant imatinib therapy. On routine follow-up visit in May 2012, local recurrence and metastasis were confirmed by imaging. He was then referred to West China Hospital in May 2012.

Beginning in May 2012, this patient received first-line imatinib orally with a dose of 400 mg/day resulting in a partial response. Disease progression occurred after the continuation of imatinib for 54 months. The patient was then treated with cytoreductive surgery combined with imatinib and showed a PFS of 7 months. New biopsy of an abdominal metastasis yielded a KIT mutation in exon 11 as well as in KIT exon 13 (V654A), confirming the clinical observation of secondary imatinib resistance (13). In May 2017, this patient received second-line sunitinib. After 11 months of treatment, sunitinib was discontinued due to disease progression. The patient refused biopsy for additional mutational analysis for personal reasons.

**Figure 1:**
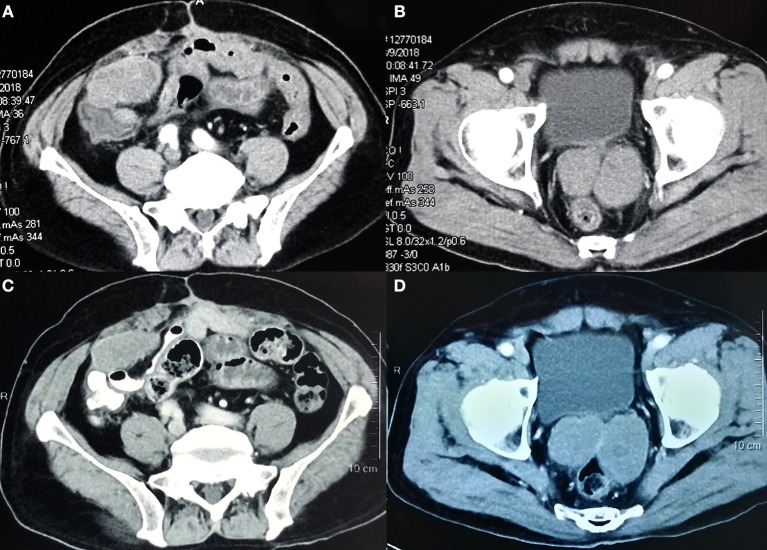
Abdominal computed tomography scans showing the metastatic lesions in the abdomen and pelvic cavity before treatment **(A,B)** to a stable disease after 8 months of treatment with apatinib **(C,D)**.

Although regorafenib had been approved for the third-line treatment of patients with advanced GISTs by China Food and Drug Administration at that time, the patient refused the agent due to the cost and budget constraints. In the meantime, there was a medical-product-donating project for apatinib that patients could get support since they were enrolled in a clinical trial. After signing informed consent, the patient was treated with apatinib 500 mg daily beginning in April 2018. Abdominal CT scans before apatinib therapy showed the metastatic lesions in the abdomen and pelvic cavity (Figures 1A,B). The drug was well-tolerated, and after 2 months of treatment, the patient had a stable disease (SD) on CT according to RECIST 1.1 (14). On routine follow-up in December 2018, the CT scan showed that the lesions were similar to the latest images, confirming a SD after 8 months of treatment with apatinib (Figures 1C,D).

During apatinib treatment, this patient developed primary side effects of hypertension (grade III) and proteinuria (grade II) according to the Common Terminology Criteria for Adverse Events (CTCAE) version 4.03 (15). Both adverse events were well-controlled with drug treatment.

On the last routine follow-up visit in December 2018, the patient is still taking apatinib as a single agent for maintenance therapy with mild toxic effects. Both clinical and imaging evaluation demonstrated no evidence of disease progression. The PFS time is more than 8 months. This study was approved by the Institutional Review Board of West China Hospital, Sichuan University (ChiECRCT-20170095). The patient gave written informed consent in accordance with the Declaration of Helsinki.

## Discussion

To date, the prognosis of patients with progression disease after the failure of imatinib and sunitinib is still poor. In this case, we administered imatinib as the first-line therapy, and PFS was 54 months. Sunitinib was administered as the second-line therapy with a PFS of 11 months. Apatinib was then administered as the third-line therapy. The tumor response was evaluated as an SD. PFS following apatinib therapy was at least 8 months. Besides, the patient tolerated apatinib well, with a satisfactory quality of life.

It is established that most (70–80%) GISTs harbor KIT mutations, resulting in ligand-independent kinase activation (4, 16). Twenty to twenty-five percent of GISTs lack KIT mutations, and of these tumors, a minority (10%) have PDGFRA mutations that are homologous to KIT mutations (prevalence of PDGFRA mutations is about 10%) (4, 16). Still, both KIT and PDGFRA mutations are missing in up to 15% of GISTs, which are called wild-type GISTs. In recent years, an increasing number of TKIs for patients with GISTs after the failure of imatinib and sunitinib have been studied. Regorafenib is the only targeted drug approved by the Federal Drug Administration for advanced GISTs after the failure of imatinib and sunitinib. In the GRID study that compared regorafenib with placebo, the results showed that oral regorafenib significantly improved PFS compared with placebo in patients with metastatic GIST after progression on standard treatments [4.8 months for regorafenib and 0.9 months for placebo; hazard ratio (HR), 0.27; 95% CI, 0.19–0.39] (17). In another Phase III study, nilotinib did not improve either survival (HR, 0.82; 95% CI, 0.64–1.15) or PFS rate as compared to best supportive care in the intent-to-treat analysis. In the *post hoc* subset analyses, in a well-defined population of true third-line patients, however, nilotinib provided significantly longer median OS (HR, 0.67; 95% CI, 0.48–0.95) (18). The RIGHT trial, a Phase III study, showed that resumption of imatinib in patients with advanced GISTs after the failure of imatinib and sunitinib significantly improved PFS (1.8 vs. 0.9 months; HR, 0.46; 95% CI, 0.27–0.78); however, it failed to improve OS (8.2 vs. 7.5 months; HR, 1.0; 95% CI, 0.58–1.83) (19).

Apatinib potently inhibited the kinase activities of VEGFR2, c-kit, and c-src, and decreased the VEGFR2, c-kit, and PDGFRβ stimulated phosphorylation at the cellular level (11). Apatinib has a clinical benefit across various cancers including breast cancer, gastric cancer, hepatocellular carcinoma, and non-small-cell lung cancer (20). Several subtypes of sarcomas have also been shown to respond to apatinib (21). Here, we report the first case of GISTs responding to apatinib. It seems that apatinib is effective in the treatment of metastatic GISTs resistant to imatinib and sunitinib.

Sunitinib and regorafenib, the second- and third-line treatment approved for GISTs, are potently targeting VEGFR in addition to KIT inhibitors. Similarly, apatinib is a potent VEGFR inhibitor apart from the KIT inhibitor. The role of VEGF in GISTs, however, has not been established. Imamura et al. suggested that angiogenesis associated with VEGF might play an important role in in the progression of GISTs (22). Several *ex vivo* studies of GIST specimens have demonstrated that microvessel density is associated with VEGF expression and closely related to the prognosis of the disease (23, 24). Recently, Verboom et al. proposed that SNPs in the genes encoding for VEGFR2 was associated with PFS in patients with advanced GISTs treated with imatinib (25). Consolino et al. suggested that VEGFR2 and VEGFR3 expression may be related to progression of imatinib-resistant GISTs, and the direct targeting of the receptors may have the potential to decrease tumor growth by the inhibition of angiogenesis (26). Thus, apatinib may have clinical benefits for patients with GISTs refractory to imatinib and sunitinib and need to be further tested in large-scale clinical trials.

## Conclusion

The present case demonstrates that apatinib provides an additional option in the treatment of patients with GISTs refractory to imatinib and sunitinib. Still, large prospective trials are required to investigate the efficacy in the treatment of the disease.

## Data Availability

No datasets were generated or analyzed for this study.

## Ethics Statement

This study was approved by the Institutional Review Board of West China Hospital, Sichuan University (ChiECRCT-20170095). The patient gave written informed consent in accordance with the Declaration of Helsinki. Written informed consent was obtained from the patient for publication of the findings of this case report.

## Author Contributions

DC and BZ conceived the idea for this case report, carried out critical interpretations, and contributed to the final version of the paper. ZC collected the data, reviewed the literature, and wrote the paper. XC prepared the figure and contributed in the revision of the literature. All the authors read and approved the final manuscript.

### Conflict of Interest Statement

The authors declare that the research was conducted in the absence of any commercial or financial relationships that could be construed as a potential conflict of interest.
